# Characterization of Lung Cancer by Amide Proton Transfer (APT) Imaging: An *In-Vivo* Study in an Orthotopic Mouse Model

**DOI:** 10.1371/journal.pone.0077019

**Published:** 2013-10-15

**Authors:** Osamu Togao, Chase W. Kessinger, Gang Huang, Todd C. Soesbe, Koji Sagiyama, Ivan Dimitrov, A. Dean Sherry, Jinming Gao, Masaya Takahashi

**Affiliations:** 1 Advanced Imaging Research Center, Harold C. Simmons Comprehensive Cancer Center, UT Southwestern Medical Center, Dallas, Texas, United States of America; 2 Department of Pharmacology, Harold C. Simmons Comprehensive Cancer Center, UT Southwestern Medical Center, Dallas, Texas, United States of America; Texas Tech University Health Sciences Center, United States of America

## Abstract

Amide proton transfer (APT) imaging is one of the chemical exchange saturation transfer (CEST) imaging methods which images the exchange between protons of free tissue water and the amide groups (−NH) of endogenous mobile proteins and peptides. Previous work suggested the ability of APT imaging for characterization of the tumoral grade in the brain tumor. In this study, we tested the feasibility of *in-vivo* APT imaging of lung tumor and investigated whether the method could differentiate the tumoral types on orthotopic tumor xenografts from two malignant lung cancer cell lines. The results revealed that APT imaging is feasible to quantify lung tumors in the moving lung. The measured APT effect was higher in the tumor which exhibited more active proliferation than the other. The present study demonstrates that APT imaging has the potential to provide a characterization test to differentiate types or grade of lung cancer noninvasively, which may eventually reduce the need invasive needle biopsy or resection for lung cancer.

## Introduction

Lung cancer is the most common cause of cancer and the leading cause of cancer-related death in both men and women in the United States. Despite the poor prognosis, when lung cancer is resected at Stage 1, five-year survival rate is as high as 70% [1]. Technical developments in computed tomography (CT) have enabled larger volume coverage with higher resolution and lower noise, and currently high-resolution CT (HRCT) is the standard imaging technique for assessing lung cancer [2] [3]. It provides excellent anatomic detail and the number of smaller lung nodules detected has increased [4]. When a noncalcified lung nodule is detected at <10 mm, follow up CT examinations to monitor the growth of the lung nodule are mandated. If the lung nodule grows, subsequent needle biopsy or video-assisted thoracoscopic resection of the lung nodule is recommended although there is still argument how accurately we can measure the growth of the lung nodule [5]. This current situation delays the start of treatment even when it is needed. In addition, cumulative radiation exposure resulting from repeated use of CT increases the risk of malignancy and the issue of radiation dose reduction, currently, draws wide attention [6], [7]. More importantly, despite advances in assessment of solitary pulmonary nodules using hemodynamic information from CT or biochemical characteristics from positron emission tomography (PET), substantial portions of solitary pulmonary nodules remain indeterminate for specific diagnosis [8]. Previous data from multi-center studies have shown that approximately 20%–50% of lung nodules removed at surgery or by needle biopsy were benign [9], [10]. These rates have been still a remaining concern [1]. These reports clearly reveal that it is imperative to develop alternative imaging methods that are radiation-free and yield second-stage characterization to distinguish benign from malignant nodule or differentiate nodular types or grades [11].

Chemical exchange transfer (CEST) has drawn considerable attention as a novel mechanism to produce contrast in MR imaging. This new method provides more detailed physiological and functional information than conventional MR imaging and has emerged in the field of molecular imaging [12], [13]. CEST contrast is achieved by applying a presaturation pulse at the resonance frequency of a slow-intermediate exchanging proton site (−NH, −OH, or metal bound water molecule) of endogenous or exogenous agents. The resulting saturated or partially saturated spin is transferred to bulk water via chemical exchange. Consequently, specific molecular information is obtained indirectly through the bulk water signal used to image tissue. The net effect of CEST is to reduce the bulk water signal intensity detected in an imaging experiment, thereby providing negative contrast in an image [14].

Amide proton transfer (APT) imaging is one subset of CEST imaging that refers specifically to chemical exchange between protons of free tissue water (bulk-water) and amide groups (−NH) of endogenous mobile proteins and peptides. It has been reported that such exchangeable protons are more abundant in tumor tissues than in healthy tissues [15]. When applied to rats implanted with 9 L gliosarcoma tumors [16], APT imaging was able to distinguish between pathology-confirmed regions of tumor and edema, which could not be accomplished using standard T1-/T2-weighted or diffusion-weighted imaging, in which the tumor border appeared diffusive. Previous reports demonstrated that CEST effects (APT ratios: APTRs) were found to increase by 3–4% in tumor compared with peritumoral brain tissue in an experimental rat glial tumor at 4.7 T [17] and human brain tumor at 3 T [18]. In the latter study in patients, the APTRs in 6 high-grade brain tumors (average 2.9±0.6% in tumor core and 2.4±0.6% in tumor periphery) were higher than those in 3 low-grade brain tumors (average 1.2±0.2%). It is presumed that these findings are consistent with work by Howe et al. [19] who found that these mobile protein concentrations were higher in tumors than in normal white matter, and increased with tumor grade in the human brain.

Unlike brain imaging, *in-vivo* MRI of the lung is challenging because of the inherent difficulties associated with properties of the organ, including respiratory and cardiac motion artifacts, severe magnetic field susceptibility arising from large air-tissue interfaces [20], [21]. In particular, the pronounced susceptibility effects in the lung may dynamically alter the magnetic field homogeneity during respiratory cycle and thus may cause shifts in the resonance frequencies of the different proton pools in the tissue. The objectives of our study are to test the feasibility of APT imaging of lung tumors in a living mouse and to investigate whether APT imaging can be a characterizing test of lung tumors. In this study, we tested the respiratory gated APT imaging under ventilation on orthotopic tumor xenografts from two malignant lung cancer cell lines: one is human lung adenocarinoma, A549, and the other is murine Lewis lung carcinoma (LLC). It is well-known that LLC is a highly malignant cancer and shows more aggressive progression than A549 after transplantation in the lung [22], [23].

## Materials and Methods

### Animal Protocol

The animal protocols were approved by the Institutional Animal Care and Use Committee at UT Southwestern Medical Center, and the experiments were performed in accordance with the National Institutes of Health Guidelines on the Use of Laboratory Animals. The orthotopic models of lung cancer in mice were introduced by the method previously reported [22]. Briefly, female athymic mice (25–30 g) were injected intravenously via tail vein with 0.5×10^6^ A549 cells (n = 6) or LLC cells (n = 6). Tumors were allowed to grow to show approximately 1.0×10^6–7^ relative light intensity on bio-luminescence imaging (BLI) and subjected to the MRI study. All animals were sacrificed and lungs were harvested after the MR imaging session.

Under anesthesia with 1.5–2% isoflurane (AERRANE, Baxter Healthcare Corporation, IL) mixed in 100% oxygen, a 1 cm non-metallic endotracheal tube (20-gauge) was placed via tracheostomy. The cannulated animal was then connected to a small animal ventilator (flexiVent, SCIREQ, Quebec, Canada) with an approximately ∼3 m tube in the supine position with the thorax centered to the center of the RF coil as previously reported [24], [25]. The animal was mechanically ventilated for constant amplitude and frequency of respiration at approximately 32 breaths/min in which inhalation (I)-to-exhalation (E) ratio (I/E) was 2/3 (I = 100 msec, E = 150 msec) and end-expiration for 1.6 s, respectively. Respiratory sensor was placed on the abdomen of the mouse. In addition, we limited the intrapulmonary pressure at end-inspiratory phase as 20 cm H_2_O so that the lung was inflated until the intrapulmonary pressure becomes 20 cm H_2_O.

### MR Imaging

**Figure 1:**
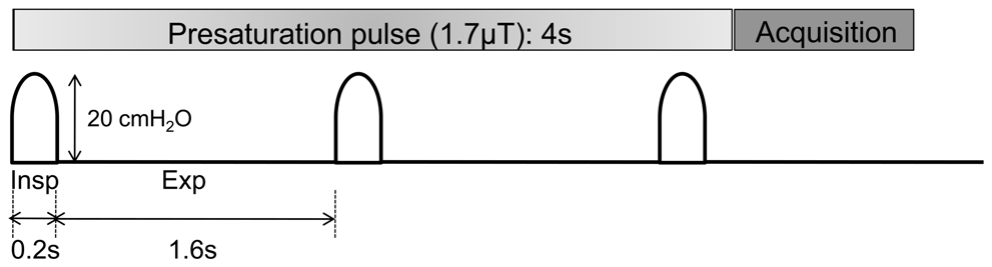
Study design for APT imaging of the mice lung using the small animal ventilator. The animal was mechanically ventilated for constant amplitude and frequency of respiration at 32 breaths/min in which inhalation and end-expiration was 0.2 s and 1.6 s, respectively. The lung was inflated until the intrapulmonary pressure becomes 20 cm H_2_O. Fast spin-echo images were obtained following a presaturation pulse (continuous-wave block pulse, B1 = 1.7 µT, duration = 4 s) in the end-expiratory phase.

MR imaging was conducted with a 7 T small animal MR system (Varian, Inc, Palo Alto, CA) with a 40 mm (I.D.) radiofrequency (RF) coil. First, low-resolution multi-slice imaging was performed on the thoracic region to confirm the location and orientation of the lung. Axial T2-weighted multi-slice images encompassing the entire lung were then obtained with a fast spin-echo sequence (repetition time/echo time = 2500/40 msec; field of view = 30×30 mm, matrix = 128×128, slice thickness = 1 mm, gapless, number of excitations = 8). On a single 1-mm-slice, delineating the tumor(s), APT imaging was performed with respiratory gating under the respiration addressed above by using a MR compatible small-animal monitoring device (SA Instruments, Inc., Stony Brook, NY). Fast spin-echo images were conducted following a presaturation pulse (continuous-wave block pulse, B1 = 1.7 µT, duration = 4 s) which was applied at 25 frequency offsets from 6 to −6 ppm with an interval of 0.5 ppm. In this system, the 4 s-presaturation pulse was applied over 2.5 respiration cycle and the image was acquired at end-expiratory phase (Fig. 1) at each offset-frequency. This fast spin-echo sequence was adapted centric k-space ordering to evoke the effect of the presaturation on image contrast. Other imaging parameters were: TR/TE = 5400/8.94 ms, FOV = 30×30 mm, echo train length = 16, matrix = 128×64 (reconstructed to 256×256), NEX = 4. A control image without the presaturation pulse was also acquired at end-expiratory phase. Total acquisition time for each animal was approximately 45 min.

### MR Imaging Data Analysis

All image data were analyzed with a program written in interactive data language (IDL; Research Systems, Inc., Boulder, CO) [18] and ImageJ (version 1.43 u; National Institutes of Health, Bethesda, MD). The definitions and terminology used in this study are equivalent to the previous papers [18], [26]. Briefly, the magnetization transfer ratio (MTR) is defined as: MTR = 1−S_sat_/S_0_, where S_sat_ and S_0_ are the signal intensities with and without presaturation pulse, respectively. In the data processing, the images obtained at 25 frequency offsets were first organized to lead the z-spectrum. Then, the z-spectrum was fitted on a pixel-by-pixel basis according to the procedure using a Gaussian fitting followed by the 12^th^-order polynomial fitting on positive and negative sides of frequency offsets, respectively, as described in previous literature [17], [18]. Subsequently, the original z-spectrum was corrected pixel-wise for the B_0_ inhomogeneity effect through the interpolation and centering of the z-spectrum. MTR asymmetry (MTR_asym_) was defined as: MTR_asym_ = MTR (+offset) – MTR (−offset) = S_sat_(−offset)/S_0_−S_sat_(+offset)/S_0_.

**Figure 2:**
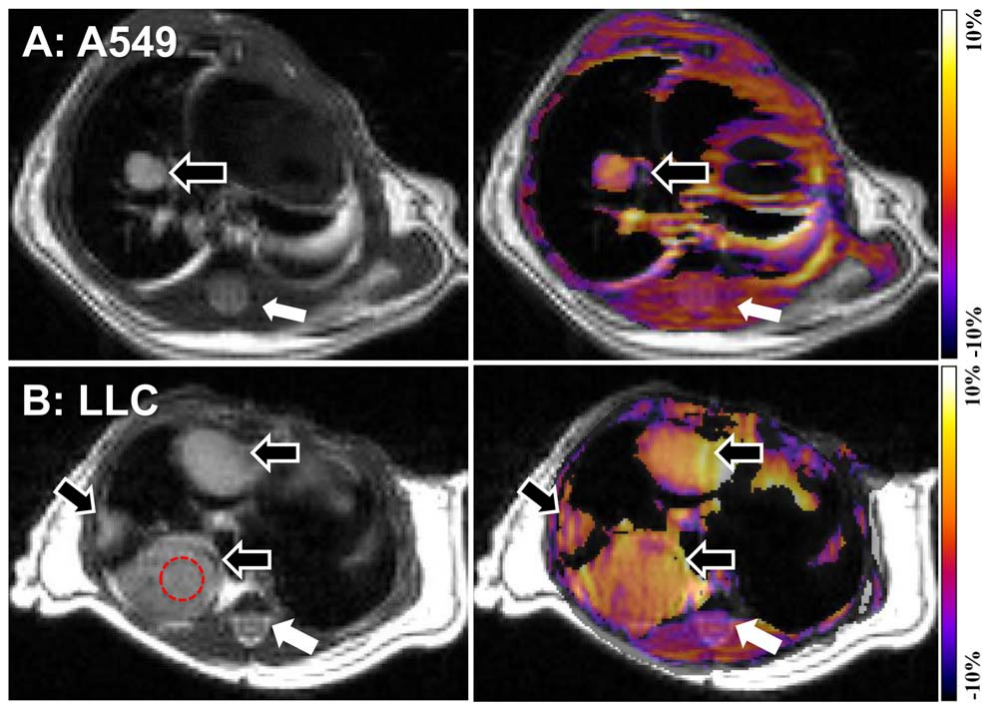
In-vivo APT imaging of lung tumors in the orthotopic mouse model. Representative T2-weighted images (left) and APT-weighted images (right, MTR_asym_ map at 3.5 ppm) of A549 (**A**) and LLC (**B**) where the tumors (open arrows) are delineated brighter than the surrounding tissues including spinal cord (closed arrows) and skeletal muscles. A typical region of interest to measure signal intensity on a tumor is demonstrated (**B**).

MTR_asym_ calculated at the offset of ±3.5 ppm reflects APT ratio (APTR) and thus the MTR_asym_ map at ±3.5 ppm is called as APT-weighted image. APT-weighted images were generated: MTR_asym_ (3.5 ppm) = MTR (+3.5 ppm) – MTR (−3.5 ppm) = S_sat_ (−3.5 ppm)/S_0_−S_sat_ (+3.5 ppm)/S_0_. To measure the local MTR_asym_, circular region-of-interests (ROIs, typical size = 0.34 mm^2^, Fig. 2B) were carefully placed on the tumors. When there were multiple tumors on the image, we averaged the results to make a representative value for the animal. The ROIs were also placed in the spinal cord for a reference. Consequently, we calculated corrected MTR_asym_ in the tumor by normalization using MTR_asym_ in the normal tissue (measured MTR_asym_ in tumor subtracted by that in spinal cord) as usual in the brain studies [17], [18] and compared the corrected MTR_asym_ between two different types of lung tumor, A549 and LLC.

### Histology

Following euthanasia, the mouse lungs were inflated in the closed chest condition by tracheal instillation of 10% phosphate buffered formaldehyde. After in situ fixation, the lungs were removed and immersed in 10% formalin. The lung tissue was embedded in optimal cutting temperature compound and flash frozen. The tissue was sectioned on a Leica 3050S cryostat at 8 µm. Pathologic slices were obtained in an axial plane and stained for hematoxylin-eosin (HE) for microscopic examination. Ki67 immunohistochemical staining was performed with the standard protocol [27]. An increase in Ki67-expression indicates an increase in mitotic cell activity and proliferation.

### Statistical Analysis

All values were expressed as mean ± standard deviation (SD). MTR_asym_ was compared between A549 and LLC groups by Student’s t-tests at each given frequency. All statistical analyses were performed by using a commercially available software (Prism 5.0, GraphPad Software, Inc., San Diego, CA), and *P*<0.05 was considered to indicate a statistically significant difference.

## Results

### APT Imaging

The animals showed approximately 1.0×10^6–7^ relative light intensity in the BLI at 5–7 weeks (A549) or 3 weeks (LLC) after the cancer cell injections, and were subjected to the APT MR imaging. On the localizer multi-slice T2-weighted images, the number, shape and size of the tumors were varied, indicating heterogeneous progression of these cancers. We selected a single axial slab (1 mm) that delineated the maximum area of the largest tumor for the further APT MR imaging in each animal. On the selected image, the average sizes (maximum diameter) of the tumors that were involved in the APT measurement were 2.0±0.5 mm in the A549 group and 2.6±1.4 mm in the LLC group where no significance was found in size between the groups (P = 0.35). All animals were successfully gated with respiration and no image was degraded by respiratory motion artifacts at any given frequency offset.

**Figure 3:**
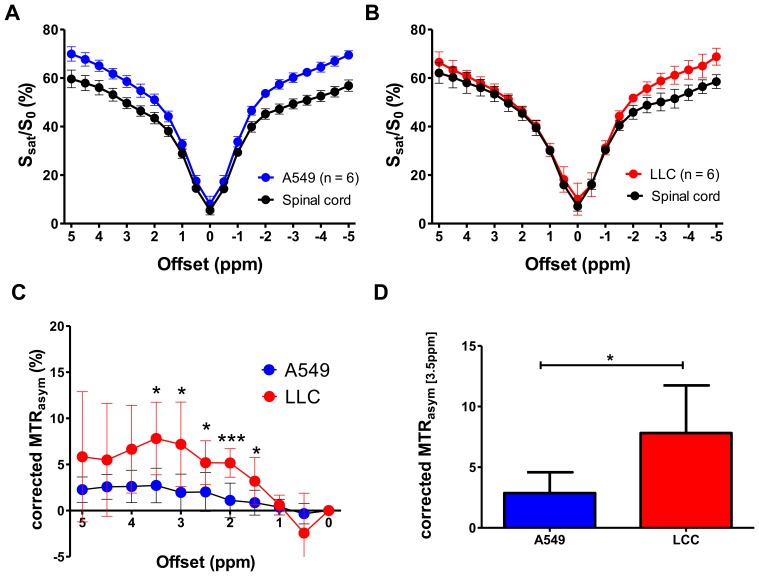
Analyses for Z-spectrum and MTR_asym_ of both types of lung tumors. Z-spectra of A549 (**A**) and LLC (**B**) tumors compared to that of spinal cord as a reference show that the LLC tumor has a larger CEST effect than A549 tumor. Corrected MTR_asym_ spectra of A549 and LLC (**C**) and corrected MTR_asym_ at 3.5 ppm (**D**) show that LLC has a larger APT effect than A549, which may be related to the malignancy of the tumors. *, *P*≤0.05; **, P≤0.01; ***, P≤0.001 by Student’s t-test.

Figure 2 shows the representative cases of both A549 and LLC groups. T2-weighted images show single or multiple solitary nodules (open arrows) in the lung in the A549 (Fig. 2A, left) or LLC (Fig. 2B, left) groups, respectively. On the APT-weighted images (MTR_asym_ map at ±3.5 ppm) of A549 (Fig. 2A, right) and LLC (Fig. 2B, right), the tumors appeared brighter than the surrounding tissues including spinal cord (closed arrows) and skeletal muscles. The z-spectrum of the LLC (n = 6) was more asymmetric than that of the A549 (n = 6) where the S_0_/S_sat_ (%) was lower at positive offsets than that at negative offsets (Fig. 3A, B). Consequently, the MTR_asym_ in LLC was consistently higher than that in A549 (at >1 ppm) and the significant differences between the groups were observed at 2 ppm (6.0±1.8% vs. 2.9±1.5%, *P* = 0.01) and at 3.5 ppm (3.2±2.9% vs. 0.7±1.3%, *P*<0.05). The corrected MTR_asym_ (Fig. 3C) between the two types of tumors became the maximum at 3–3.5 ppm and showed significant difference at 3.5 ppm (7.8±3.9% vs. 2.7±1.9%, *P*<0.05, Fig. 3D).

### Histology

**Figure 4:**
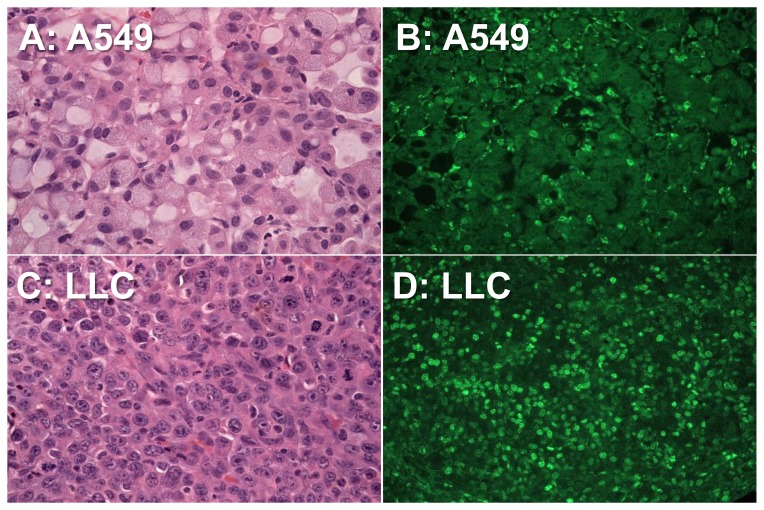
Micrographs of the A549 tumor and LCC tumor. Hematoxylin-eosin staining (original magnification×400) demonstrates that LCC (**C**) have higher cell density and larger cell nuclei compared to A549 (**A**). Ki-67 staining (original magnification×200) reveals larger fraction of positive cells seen in LCC (**D**) than in A549 (**B**). This indicates the presence of a larger number of cells in active phases of the cell cycle (G_1_, S, G_2_, and mitosis) and thus the aggressive nature of LCC.

Figure 4 demonstrated the typical microphotographs stained by HE and Ki67 in both types of tumors. LCC (Fig. 4C) shows higher cell density and larger cell nuclei compared to A549 (Fig. 4A) in HE staining. Ki-67 staining reveals larger fraction of positive cells, which is found in LCC (Fig. 4D) more than in A549 (Fig. 4B). This indicates that LLC possesses a larger number of cells in active phases of the cell division cycle (G_1_, S, G_2_, and mitosis) and thus it is more active proliferation than A549.

## Discussion

In the present study, we demonstrated the feasibility of the *in-vivo* APT imaging of lung tumors in the orthotopic mouse model and that the method quantitatively distinguished two different types of lung tumors. Our major concern was whether the long (4 s) continuous-wave preparation pulse over several respiratory cycles could saturate the amide proton signal exchanging with that of bulk water homogenously at each frequency offset over the thorax including tumors. With a simple respiratory gating, actual TR depends upon respiratory rate that often changes under free breathing, leading modulation of the MR signal intensity. More importantly, alteration of respiratory frequency and amplitude causes different excursion of the tumors in the lung [28], [29] where the tumors might be exposed to different level of field inhomogeneity or effect of presaturation pulse in each signal acquisition. To minimize these effects, we utilized a small animal ventilator to introduce constant frequency and amplitude of the respiration so that the preparation pulse and subsequent acquisition were implemented at a fixed timing completely during breathing (Fig. 1). We also selected a centric k-space ordered fast spin-echo sequence as it is less sensitive to susceptibility effect. Under these conditions, the obtained z-spectra exhibited reasonably small variations among the animals at any given frequency offset (Fig. 3A and B) and could distinguish the different types of the tumors in the lung (Fig. 3C and D).

The measured asymmetry curves in both types of tumors show that the MTR_asym_ increased from the resonance respect to bulk-water (0 ppm) and reached the maximum at 2 ppm and then reduced at higher offset (2–5 ppm). This is consistent with the MTR_asym_ observed in the brain tissues [16], [17], [30]. It was reported in a NMR study that the amide protons of mobile protein/peptide side-chains (Gln, Asn) and backbones resonate at 6.8 ppm (2 ppm downfield from the water signal) and in the 8.2–8.4 (3.5 ppm downfield from the water signal) ppm range, respectively [31]. These are also observed in the normal tissues, and thus the background MT effect is not symmetric with respect to the water resonance in the frequency range of aliphatic (2–5 ppm). This inherent asymmetric MT effect, the intramolecular and intermolecular nuclear Overhauser effects (NOE) of aliphatic protons of mobile macromolecules and metabolites contaminate the measured CEST (APT) effect in the observed MTR_asym_ [16], [32]. To eliminate these effects, the magnitude of APTR is often determined from the difference of MTR_asym_ at the lesion and the contralateral regions in the previous brain studies [15], [17]. Our study in the orthotopic lung cancer model did not have such a reference tissue since contralateral normal lung parenchyma has almost no signal. Hence, we attempted to use the spinal cord as a reference tissue (Fig. 3A and B). The MTR_asym_ in the spinal cord was −4 to −1%, which was approximately same level and consistent with those reported in the normal brain tissues [16], [17]. The corrected MTR_asym_ (the MTR_asym_ subtracted by that in the spinal cord) in both groups increase from 1 ppm and reached the maximum at 3.5 ppm and showed statistical significance between the A549 and LLC groups at 1.5–3.5 ppm (Fig. 3C). The corrected MTR_asym_ at 3.5 ppm could discriminate between two types of tumors; it was higher in LLC than in A549 (Fig. 3D).

Previous study demonstrated that BLI offered a simple and rapid technique for assessing tumor growth in rodent models of brain tumor noninvasively, which correlated well with MRI [33]. BLI was also demonstrated to be a reliable approach for monitoring the growth of human lung cancer cells in orthotopic murine models [23]. Therefore we used BLI to decide the timing to implement the APT imaging in each animal. The timings to show 1.0×10^6–7^ relative light intensity after the cancer cell injection varied and were somewhat different between the groups (5–7 weeks for A549 and 3 weeks for LLC). Although it is difficult to assure whether the developmental stage was equivalent between the groups, the size of tumors that we measured APT was not different between the groups (P = 0.14). It was revealed that the LLC showed denser cellularity and more active proliferation in the histological examination (Fig. 4). Our results were consistent with the result referring to that LLC shows more aggressive profile than A549 [22], [23]. Since there is no orthotopic animal model of benign pulmonary nodule, we previously measured APTR with identical imaging protocol in several types of cell lines in vitro [34]. In the study, the APTR in a normal lung cell line (HSAEC1-KT) was much lower than that of the malignant tumor cell lines (A549 and H1299). Further, the APTR in the normal cell line markedly increased after the cell was driven oncogenesis. Based on these results, we believe that the observed corrected APTR between the groups could reflect different tissue concentration of mobile proteins/peptides. Thus, we postulate that the results would reveal the potential of APT imaging for characterization of tumoral types which possess different histological features, especially between benign and malignant. To elucidate whether APT imaging could differentiate among specific types of lung tumors, e.g. among non-small cell lung carcinomas or between non-small cell lung carcinoma and small cell lung carcinoma, further studies are necessary to quantify relation between APTR and ‘malignancy’ using several different types/grades of lung tumors.

Although we did not quantify field inhomogeneity that may alter during respiration in the lung, our results indicated that APT imaging is feasible to quantify lung tumors in the moving lung when the preparation pulse and acquisition were completely synchronized with a constant respiration. As it is difficult to control respiration in patients, we should further evaluate how motion effects on APT signal and how we can overcome this issue. We have demonstrated that respiratory gating would help to implement the CEST imaging in human kidney [35]. If the method could be executed under breath hold (∼20 s) with a fast imaging sequence such as key-hole CEST [36], this will also help to minimize the difficulties relating to respiratory motion. These ideas in conjunction with motion management paradigms [37] may advance clinical translation of the method in the lung.

As it is still confounded to extract APT effect, the measurement might be improved by robust ideas for future studies. First, z-spectra can be more precisely interpolated by increased number of frequency offsets actually measured, in particular for the frequency ranges showing the peaks for APT (±2–5 ppm) and bulk water (±∼1 ppm). Adequate number of offsets should be decided with consideration for total scan time, in particular for human study. The shift of bulk water peak by B_0_ inhomogeneity is more effectively estimated and corrected by collecting B_0_ map [38]. If the target chemical shift is closer to water resonance and the water peak in the z-spectrum is broader (direct water saturation effect is prominent), B_0_ correction can be effective by using WASSR (water saturation shift referencing) method [39] although it was not the case in the current study. To reduce effects from background MT and field inhomogeneity, Scheidegger et al. reported APT-SAFARI (saturation scheme-saturation with frequency alternating RF irradiation) with a pulsed off-resonance saturation module followed by a single-slice EPI read-out in which ±3.5 ppm are simultaneously saturated [40]. To have reference tissue to lung tumors discussed above, development of CEST sequence in conjunction with ultra-short echo time (UTE) MRI [24], [25] or SWIFT (sweep imaging with Fourier transform) -CEST [41] that enables producing MR signal from lung parenchyma may be effective.

In summary, the present study demonstrates that APT imaging is feasible and has the potential to provide cancer-specific imaging to characterize types or grade of lung cancer noninvasively. The method can be a characterizing test of lung tumors and may eventually reduce the need invasive needle biopsy or resection. We may be able to decide appropriate treatment, start early treatment and monitor the progression of tumor or to evaluate response to therapy.
